# Pt Single Atom Deposition by Direct Current Sputtering in the Gas‐Scattering Regime—A Simple Approach to Controlled Single Atom Loading

**DOI:** 10.1002/cssc.202500489

**Published:** 2025-05-15

**Authors:** Xin Zhou, Jihyeon Kim, Hyesung Kim, Sani Zahra, Patrik Schmuki

**Affiliations:** ^1^ Department of Materials Science WW4‐LKO Friedrich‐Alexander‐University of Erlangen‐Nuremberg Martensstrasse 7 91058 Erlangen Germany; ^2^ Regional Centre of Advanced Technologies and Materials Šlechtitelů 27 78371 Olomouc Czech Republic

**Keywords:** hydrogen generation, photocatalysis, platinum, single‐atom catalysis, sputter deposition

## Abstract

Supported single atoms represent a new frontier in many catalytic fields, and noble metal single atoms in particular have been reported to be a highly effective cocatalyst in photocatalysis or electrocatalysis. Herein, it is described that direct current sputtering of Pt, when operated under plain gas‐scattering conditions, can be leveraged for single atom deposition on various substrates (herein TiO_2_ and graphene are used as examples). The approach allows a uniform single atom deposition with a high level of control over single atom density and loading amount on both surfaces. Such Pt single atoms on TiO_2_ can be used directly (without further treatments) as cocatalysts for photocatalytic H_2_ generation. Remarkably, single atom loading and H_2_ generation activity correlate linearly over a wide range of Pt loading (0.16–1.41 at%). The findings not only open a new avenue for the synthesis and use of single atoms in photocatalysis but also highlight the use of gas‐phase scattering as a simple, scalable, and versatile approach for single‐atom catalyst fabrication.

## Graphical abstract

Highly controlled Pt single atom deposition (SA) is achieved by gas‐phase scattering sputtering on various substrates. This method enables soft Pt single atom loading and uniform SA distribution. In the example, it is shown that how Pt SA loading on TiO_2_ can be established and used for photocatalytic H_2_ generation.

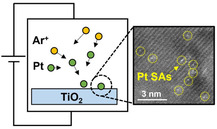

## 1 Introduction

Single atom (SA) catalysis—namely, SAs loaded on a suitable substrate—has emerged as a transformative concept in heterogeneous catalysis, offering both economic and functional advantages. As a result, SA catalysts have been extensively investigated in classical heterogeneous reactions, including oxidation, hydrogenation, and CO_2_ reduction.^[^ ^1^, ^2^, ^3^, ^4^ ^]^ More recently, SAs have also attracted significant interest in photocatalysis, where they are explored as cocatalysts for reactions of considerable scientific and technological importance.^[^ ^2^, ^5^, ^6^ ^]^

A most prominent (widely studied) example of SAs use is in photocatalytic H_2_ production from aqueous solutions, with or without sacrificial agents.^[^ ^5^, ^7^, ^8^ ^]^ At the core of any photocatalytic reaction is a semiconductor that, when illuminated with super‐bandgap (solar) light, generates mobile electron–hole pairs. These charge carriers migrate to the semiconductor surface, where they trigger oxidation and reduction reactions in the environment.^[^ ^9^, ^10^, ^11^, ^12^, ^13^ ^]^ Photocatalytic H_2_ production relies on the reduction of H^+^ or H_2_O to dihydrogen. However, this process is often hindered by slow charge transfer kinetics. To address these challenges, semiconductors are commonly decorated with cocatalysts, namely, noble metal nanoparticles (NPs, e.g., Pt, Pd, Rh) which accelerate electron transfer and significantly improve the reaction rate for H_2_ evolution.^[^ ^14^, ^15^, ^16^, ^17^ ^]^

Classically, noble metals are placed on the semiconductor as metallic NPs of a few nm in diameter. Here, shifting from metal NPs to SA‐based cocatalysts can offer drastic advantages, including maximum atom utilization (making the use of Pt as a cocatalyst economically and sustainably feasible).^[^ ^1^, ^5^ ^]^ Moreover, SAs in such a context can show unexpected selectivity and activity.^[^ ^5^, ^18^, ^19^, ^20^, ^21^ ^]^ There are many methods to synthesize SAs either by wet or dry approaches.^[^ ^22^ ^]^ Traditionally, atomic‐scale precision in deposition has been achieved using techniques such as atomic layer deposition, chemical vapor deposition, and molecular beam epitaxy.^[^ ^23^, ^24^, ^25^, ^26^, ^27^ ^]^ While they are effective ways to synthesize SAs, these methods often suffer from precursor limitations, high processing costs, and substrate compatibility constraints. In contrast to these classic methods, SA catalysts are meanwhile widely synthesized using solution‐based deposition techniques, which however require multiple preparation steps, complex precursor chemistries, and postdeposition treatments that often lead to metal aggregation.^[^ ^7^, ^28^ ^]^

In this study, we introduce a novel, versatile, and scalable approach for Pt SA deposition using Pt direct current (DC) sputtering exploiting the gas‐phase scattering (GPS) regime, i.e., conditions that utilize gas‐phase transport and gas‐scattering to achieve highly uniform SA dispersion.^[^ ^29^, ^30^ ^]^

To allow for defined studies, we use TiO_2_ thin films and graphene sheets as substrates that are transmission electron microscopy (TEM)‐transparent and thus allow for direct high‐angle annular dark‐field scanning transmission electron microscopy (HAADF‐STEM) observation, as well as characterization with usual surface science tools, such as X‐ray photoelectron spectroscopy (XPS) or high‐resolution scanning electron microscope (SEM), etc. To demonstrate the direct use of such SA decorated surfaces, we decorate flat anatase (thin‐film) surfaces with Pt SAs using GPS deposition and evaluate their performance in photocatalytic H_2_ production. We find that defined SA deposition is possible over a wide concentration range from 0.16 at% to 2.49 at% and a H_2_ production that linearly scales with the SA density.

Most importantly, we show that the GPS approach enables stable and defined SA decoration, providing an effective and scalable strategy for the synthesis of SA‐functionalized surfaces.

## 2 Results and Discussion

**Figure 1:**
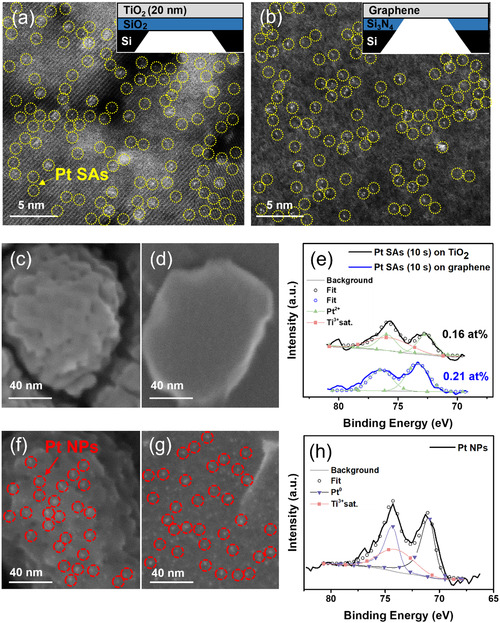
a) HAADF‐STEM images and (inset) sketch of the sample stage with thin TiO_2_ layer deposited on SiO_2_. b) Graphene on Si_3_N_4_‐Si TEM gird. c,d) After Pt GPS deposition (10 s). SEM images. e) XPS Pt 4f spectra of sputtered Pt SAs (10 s) on TiO_2_ and graphene support. f) SEM images of Pt NPs on TiO_2_. g) Graphene. h) XPS Pt 4f spectra of Pt NPs on TiO_2_.

For a most direct assessment of Pt SA deposition on different substrates, we used photolithographically defined TEM grids as described previously^[^ ^28^, ^31^ ^]^ and as illustrated in the insets of **Figure** **1**a,b that either is a window of SiO_2_ coated with ≈20 nm anatase TiO_2_ (Figure 1a) or alternatively a free‐standing single layer graphene sheet suspended over Si_3_N_4_ openings (Figure 1b). Onto both of these layer surfaces, we sputter‐deposited in a first experiment Pt for 10 s using GPS conditions (see experimental details). The HAADF‐STEM images shown in Figure 1a,b confirm that these ultrathin TiO_2_ or graphene layers allow for direct atomic‐scale imaging of Pt SAs (yellow circles) and for an evaluation of their distributions. Evidently, 10 s deposition leads on both substrates to a uniform dispersion of SAs and the evaluation of several high‐angle dark field images yields a density of dispersed single Pt atoms of ≈1.5 × 10^5^ atoms μm^−2^ for TiO_2_ and 1.8 × 10^5^ atoms μm^−2^ for graphene substrates. Note that this difference between the substrates is well within the uncertainty of the experiment and data evaluation. In SEM images (examples are shown in Figure 1c,d), no distinct Pt NPs can be found on either substrate. This shows that over large surface areas no significant NP formation occurs and supports uniform SA dispersion, where isolated atoms remain below the resolution limit of conventional field‐emission scanning electron microscopy (FE‐SEM) imaging.

Further confirmation of large‐scale Pt SAs deposition is revealed through XPS analysis on both the TiO_2_ and graphene surfaces. For the TiO_2_ support, the Pt 4f spectra (Figure 1e) exhibit a distinct doublet of a Pt 4f_7/2_ peak at 72.5 ev and a Pt 4f_5/2_ at 75.9 eV while for the graphene support a doublet of a Pt 4f_7/2_ peak at 73.2 ev and a Pt 4f_5/2_ at 76.4 eV is obtained–these peak positions are well in line with previous reports of Pt SAs placed on TiO_2_ or graphene supports using other techniques.^[^ ^7^, ^32^ ^]^ The peak positions correspond to a formal oxidation state of Pt (*δ^+^)* ≈2, which indicates that deposited Pt atoms are chemically interacting with their surface rather than forming aggregated metallic clusters. The Pt loading from XPS is very similar for the two supports, with 0.16 at% and 0.21 at% for TiO_2_ and graphene, respectively (Figure 1e).

For comparison, we decorated these TiO_2_ or graphene surfaces with Pt NPs of some nm using classic photo‐deposition (TiO_2_) and electrochemical reduction (graphene) as described in the experimental section. FE‐SEM of these samples show distinct Pt NPs approx. 2 nm in size (Figure 1f,g, red circles). Such NPs lead in XPS spectra (see Figure 1h) to a Pt 4f doublet at Pt 4f_7/2_ ≈71.3 eV and Pt 4f_*5/*2_ ≈74.5 eV, corresponding to metallic Pt^0^—well in line with literature data on bulk metal Pt.^[^ ^33^ ^]^

**Figure 2:**
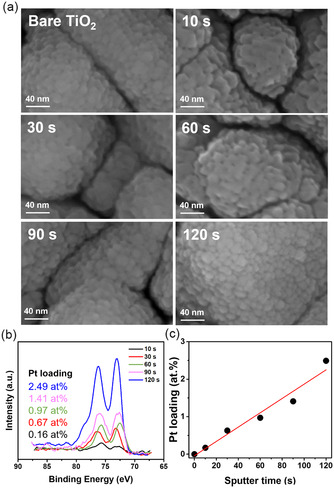
a) SEM images. b) XPS Pt 4f spectra. c) The obtained Pt loading (at%) of Pt SAs on TiO_2_ with different Pt sputter time (10–120 s).

These findings show that the combination of XPS and SEM allows to infer (indirectly) the uniform presence of Pt SAs. This is particularly important for samples that do not allow for a direct HAADF‐STEM observation, namely, thicker samples as typically used in photoabsorption‐related applications. Photocatalytic studies of TiO_2_ layers require a light absorber thickness >100 nm to obtain reliable H_2_ production data. Therefore, we used 200 nm thick anatase layers for photocatalytic H_2_ production experiments, and assessed different SA deposition via SEM and XPS. **Figure** **2**a displays SEM images of 200 nm‐thick sputtered anatase TiO_2_ layers on fluorine‐doped tin oxide (FTO) glass after Pt SA deposition via GPS sputtering under identical conditions as for the TEM‐transparent layers in Figure 1. To vary the Pt loading, we then varied the GPS deposition times to 30 s, 60 s, 90 s, and 120 s. The corresponding SEM images in Figure 2a confirm for all samples the absence of FE‐SEM visible Pt aggregation to NPs. More importantly, XPS analysis of the Pt species on these 200 nm‐thick anatase TiO_2_ layers (Figure 2b) reveals the same Pt speciation, with Pt 4f peaks at the same positions as for the 10 s deposited sample in Figure 1 i.e., Pt 4f_7/2_ at ≈72.5 eV – confirming the successful decoration of the TiO_2_ surfaces with SA Pt species up to deposition times of 120 s. With increasing deposition time, the surface concentration of Pt derived from XPS is 0.16 at% for 10 s, 0.67 at% for 30 s, 0.97 at% for 60 s, 1.41 at% for 90 s, and 2.49 at% for 120 s, i.e., the loading linearly increases with deposition time (Figure 2c).

**Figure 3:**
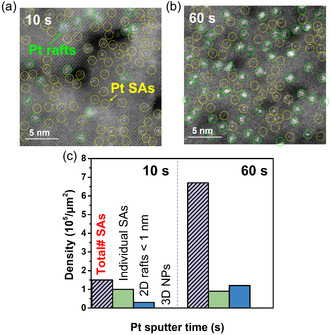
a,b) HAADF‐STEM images. c) Statistic evaluation of Pt on TiO_2_ with Pt sputter time of 10–60 s.

To additionally assess the SA distribution at a higher loading, we acquired HAADF‐STEM for a sample deposited for 10 s and 60 s on the 20 nm thick TiO_2_ surface (TiO_2_/Pt GPS, **Figure** **3**a,b). For this sample, evidently, to some degree aggregation to 2D rafts has taken place. Except for Pt loading determined by XPS (Figure 2b,c), we counted the number of Pt atoms present in HAADF‐STEM, distinguishing i) the total number of atoms, ii) the number of clusters present (clear agglomeration of several SAs to 2D rafts <1 nm), and iii) 3D particles. For statistical evaluation, the Pt SA density is calculated from several STEM images taken at different locations. We counted SA‐assemblies (rafts) as agglomerates if the Pt‐Pt distance is less than a few atom diameters and grouping occurs. An evaluation of the distribution of Pt (SAs and rafts) for the 10 s sample and the 60 s sample is shown in Figure 3c. For TiO_2_/Pt GPS (10 s), a density of individual Pt SA (yellow circles) and 2D raft (green circles) is ≈1.0 × 10^5^ atoms  μm^−2^ and ≈0.3 × 10^5^ rafts μm^−2^, respectively while ≈0.9 × 10^5^ atoms μm^−2^ and ≈1.2 × 10^5^ rafts  μm^−2^ are obtained for TiO_2_/Pt GPS (60 s). Note that Pt 3D NPs are still not observed even at higher deposition levels (120 s).

Total density of Pt atoms on TiO_2_ after GPS 10 s and 60 s is 1.5 × 10^5^ atoms μm^−2^ and 6.7 × 10^5^ atoms μm^−2^, respectively, i.e., the total amount of atoms for the 60 s sample is about 5 times higher than for the 10 s sample, well in line with the XPS data of 0.16 at% for the 10 s and 0.97 at% for the 60 s sample, and in accord with the linear dependence of deposition time and Pt uptake. This vice versa shows that the HAADF‐STEM images are representative of the Pt distribution on the overall surfaces of the 10 s and 60 s samples. A further remarkable point is that the agglomeration visible in the 60 s sample (mainly the number of 2D rafts increased) does not lead to a noteworthy shift in the XPS peak position. This indicates that in these rafts, the Pt‐Pt interactions are not dominant, but rather still the support interactions dominate (i.e., the nature of individual Pt SAs is preserved).

**Figure 4:**
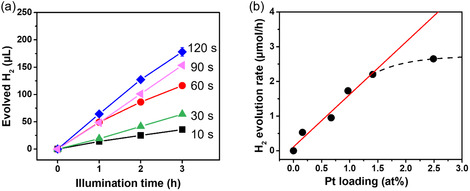
a) Photocatalytic H_2_ evolution of sputtered Pt SA on TiO_2_ layers. b) Photocatalytic H_2_ evolution rate as a function of Pt loading.

The 200 nm thick anatase TiO_2_ layers were then investigated for photocatalytic H_2_ evolution performance under UV irradiation (365 nm, 65 mW cm^−2^) in MeOH 50 vol% aqueous solution. **Figure** **4**a shows the H_2_ production over irradiation time. For all the samples, the H_2_ evolution amount increases linearly over irradiation time (Figure 4a), i.e., from the linear slope of these curves a H_2_ evolution rate can be determined. Figure 4b shows the H_2_ production rate versus the Pt loading for the different samples. Clearly, a linear increase of the H_2_ production rate with an increased Pt SA loading can be observed. The fact that the rate increases linearly with the Pt loading from 0.16 at% to 1.41 at% is well in line with the XPS data, i.e., that a similar cocatalyst species is permanently increased without inducing change in the cocatalytic mechanism or rate‐determining step. This shows that SAs, although they seem increasingly mildly agglomerated to 2D rafts are similarly (increasingly) contributing to the enhancement of the reaction. Only for Pt surface concentration higher than 1.41 at%, a saturation of the catalytic efficiency is observed.

In other words, the observed linear correlation between Pt loading (0.16–1.41 at%) and H_2_ production rate suggests that the Pt SAs maintain their catalytic activity even as their density increases. Although some mild agglomeration into **2D rafts** occurs, the Pt SAs, whether isolated or forming small rafts, continue to contribute effectively to the enhancement of photocatalytic H_2_ evolution. Overall, this study demonstrates that GPS sputtering enables highly controlled deposition of active SAs onto various substrates. Given the expanding role of SA catalysts in diverse scientific fields, this approach offers significant implications for material science, catalysis, and beyond.

## 3 Conclusion

This study demonstrates a simple, scalable, and highly controlled approach for SA Pt deposition using DC sputtering in the gas‐scattering regime. The method ensures a uniform distribution of Pt SAs on TiO_2_, achieving precise control over atomic‐scale loading without requiring complex precursor chemistries or postdeposition treatments. The resulting Pt SA‐decorated TiO_2_ films exhibit efficient and stable photocatalytic H_2_ production, with a direct correlation between Pt loading and activity. Even at increased loadings, Pt SAs remain well‐dispersed, with only mild agglomeration into 2D rafts, which still actively contribute with a SA characteristic to the catalytic process. This GPS sputtering approach provides a cost‐effective and versatile strategy for SA catalyst fabrication, extending its potential applications to other SA applications, thus contributing a promising alternative route to the next generation of atomically precise catalysts.

## 4 Experimental Section

4.1

4.1.1

##### Preparation for Reactive Magnetron Sputtering

TiO_2_ films were deposited on fluorine‐doped tin oxide (FTO) by reactive DC magnetron sputtering (DC‐MS). Beforehand, the FTO glasses (7 Ω m^2^, Pilkington) were sonicated sequentially in acetone, ethanol, and deionized (DI) water for 15 min, and then dried by nitrogen stream. Afterward, they were inserted into the sputter load‐lock (LL), whose vacuum was then equalized to that of the main sputter chamber (SC, Createc – SP‐*P*‐US‐6M‐3Z). The cleaned substrates were then transferred to a sample holder in the SC for the following sputter processes.

In advance of compact oxide deposition on the substrates, a titanium target (5”, 99.995%, dia. 127 mm × 8 mm, HMW – Hauner GmbH & Co. KG) conditioning was carried out under pure Ar atmosphere to eliminate the passivating oxide layer caused by previous sputtering. Preliminarily, the base pressure ($P_{b}$) was adjusted to ≈1.5 ×10^−7^ mbar. Afterward, 100% turbo‐molecular pump speed was decreased to 20%. Under the reduced pump speed condition, Argon gas (4 sccm) was introduced into the main SC. When the working pressure ($P_{w}$) was stabilized at ≈1.4 ×10^−3^ mbar, DC power was supplied to the magnetron and increased from 50 W to 500 W for 10 min with a closed shutter.

##### Fabrication of TiO_2_/Ti/FTO Samples

Particularly, a thin layer of Ti metal was deposited by the 5” Ti target prior to TiO_2_ compact oxide layer deposition. The substrate holder was first set to rotate (50 rpm) for uniform deposition. Subsequently, 150 W of DC power was applied for 10 s to accomplish the deposition of Ti interlayer on FTO glasses without substrate heating.

After all the pretreatments, main TiO_2_ layers were then deposited by reactive DC‐MS method with the conditioned 5” Ti target. Argon gas (10 sccm) and oxygen gas (5 sccm) were steadily flowing into the SC to the point where $P_{w}$ was stabilized at ≈6.7 × 10^−3^ mbar. Partial pressure of the oxygen gas ($P_{O_{2}}$) was fixed at 33.3%. Mass flow controllers (MFC, MKS Instruments, Inc.) precisely controlled flow rate and partial pressure of the gases. The distance between the titanium target and the FTO substrates was kept at 115 mm. Sputter deposition was implemented at room temperature (≈18 °C) under the circulating cooling water system for magnetron cooling. The sputtering process was performed at 500 W for optimized times. For uniform film thickness, the substrate holder was continuously rotating at 20 rpm during the deposition. The deposited layers were finally annealed at 450 °C for 1 h in an air tubular furnace.

##### Pt Single Atom Deposition on TiO_2_/Ti/FTO and Graphene Sheet

Pt SA deposition was carried out by sputter deposition (Leica EM SCD 500). Sputter deposition was done at a pressure of 10^−2^ mbar and a current of 16 mA with Pt target (dia./thickness 54 × 0.2 mm – HMW Hauner GmbH & Co.KG). Deposited Pt loading was controlled by sputter time from 10 s to 120 s.

In our approach, Pt SAs deposition relies on GPS. In this process, sputtered Pt atoms do not travel directly to the substrate but rather undergo gas‐phase collisions with neutral argon or other gas molecules in the chamber. These collisions lead to a diffuse, isotropic distribution of Pt, resulting in a highly uniform deposition of SA across the TiO_2_ surface. By varying the sputtering duration, we controlled Pt surface coverages, allowing us to investigate different atomic‐scale loadings.

##### Pt NP Deposition on TiO_2_/Ti/FTO

Pt NPs were deposited on TiO_2_ and graphene by photodeposition and electrodeposition, respectively. For Pt NPs deposition on TiO_2_, 2 mm of Pt precursor (H_2_PtCl_6_ × H_2_O, 40% Pt, Metakem) was prepared in 10 mL of methanol 50 vol% aqueous solution (MeOH, ≥99.9%, Carl Roth) and purged with Ar gas for 15 min. TiO_2_ substrate was then placed in a quartz reactor containing Pt precursor solution and illuminated with UV light (LED, λ = 365 nm and 65 mW cm^−2^) for 1 h. After photodeposition, Pt NPs deposited TiO_2_ were washed with deionized water and dried with N_2_ gas.

##### Characterization

HAADF‐STEM was carried out by a high‐resolution transmission electron microscope (HRTEM, FEI Titan G2 60–300). Surface and cross‐section morphologies of samples were investigated by FE‐SEM (S‐4800, Hitachi). The composition and chemical state of samples were analyzed by XPS (PHI5600). The peak deconvolution was carried out by MultiPak software and all XPS spectra were calibrated by shifting the spectra to a binding energy of Ti2p of 458.5 eV and C1s of 284.8 eV.

For the hydrogen evolution measurements, a quartz reactor containing 10 mL 50 vol% aqueous methanol solution was used. Also, after purging for 15 min with Ar and then being sealed, a series of samples were irradiated with an LED (365 nm, 65 mW cm^−2^). The photocatalytic H_2_ was determined by a gas chromatograph (GCMS‐QO2010SE, SHIMADZU) with a thermal conductivity detector.

## Conflict of Interest

The authors declare no conflict of interest.

## Data Availability

The data that support the findings of this study are available in the supplementary material of this article.
